# CCN1 Induces **β**-Catenin Translocation in Esophageal Squamous Cell Carcinoma through Integrin **α**11

**DOI:** 10.5402/2012/207235

**Published:** 2012-05-31

**Authors:** Jianyuan Chai, Cristina Modak, Yi Ouyang, Sing-Yung Wu, M. Mazen Jamal

**Affiliations:** ^1^Laboratory of GI Injury and Cancer, VA Long Beach Healthcare System, Long Beach, CA 90822, USA; ^2^Division of Gastroenterology, Department of Medicine, University of California, Irvine, Orange, CA 92868, USA; ^3^Pathology and Laboratory Medicine Service, VA Long Beach Healthcare System, Long Beach, CA 90822, USA; ^4^Diagnostic and Molecular Medicine Service, VA Long Beach Healthcare System, Long Beach, CA 90822, USA

## Abstract

*Aims*. Nuclear translocation of *β*-catenin is common in many cancers including esophageal squamous cell carcinoma (ESCC). As a mediator of Wnt signaling pathway, nuclear *β*-catenin can activate many growth-related genes including CCN1, which in turn can induce *β*-catenin translocation. CCN1, a matricellular protein, signals through various integrin receptors in a cell-dependent manner to regulate cell adhesion, proliferation, and survival. Its elevation has been reported in ESCC as well as other esophageal abnormalities such as Barrett's esophagus. The aim of this study is to examine the relationship between CCN1 and *β*-catenin in ESCC. *Methods and Results*. The expression and correlation between CCN1 and *β*-catenin in ESCC tissue were examined through immunohistochemistry and further analyzed in both normal esophageal epithelial cells and ESCC cells through microarray, functional blocking and *in situ* protein ligation. We found that nuclear translocation of *β*-catenin in ESCC cells required high level of CCN1 as knockdown of CCN1 in ESCC cells reduced *β*-catenin expression and translocation. Furthermore, we found that integrin *α*_11_ was highly expressed in ESCC tumor tissue and functional blocking integrin *α*_11_ diminished CCN1-induced *β*-catenin elevation and translocation. *Conclusions*. Integrin *α*_11_ mediated the effect of CCN1 on *β*-catenin in esophageal epithelial cells.

## 1. Introduction

While the incidence of most cancers is declining, esophageal cancer has been continuing its march as the fastest growing malignancy in the Western world [1]. Nearly 95% of esophageal cancer is esophageal squamous cell carcinoma (ESCC), whose five-year survival rate is approximately 15%, with most patients dying within the first year of diagnosis. 

Normal esophagus is covered on the lumen side by stratified squamous epithelium in which cells are connected to each other through multiple intercellular locks, such as tight junctions, adherens junctions and desmosomes. one of the components of adherens junctions is *β*-catenin. In normal epithelium, *β*-catenin is phosphorylated by glycogen synthase-3*β* and casein kinase I to keep its level low, and any loose *β*-catenin is degraded and recycled through ubiquitination. However, if for any reason *β*-catenin is unphosphorylated, it can accumulate in the cytoplasm or move into the nucleus where it becomes a mediator of Wnt signaling pathway, which could lead to transcriptional activation of several oncogenes and promote cancer [2, 3]. For this reason, *β*-catenin translocation has been found in many types of cancers including ESCC.

CCN1 (formerly known as Cyr61 or IGFBP10) is the first member of the CCN family [4], a group of matricellular proteins that share a multimodular domain structure and have been recognized by more and more people as major players in global control over cellular activities in tissues and organs [5, 6]. As secreted molecules, CCN proteins bridge the functional and physiological gap between structural macromolecules in the extracellular matrix and soluble molecules such as growth factors and cytokines. They signal through different integrin receptors in a cell- and function-dependent manner. Each combination of *α* and *β* integrin heterodimer has its own binding specificity and signaling properties, which are further diversified through alternative splicing and/or posttranslational modifications [7]. To date, almost all of the known activities of CCN1 are mediated through a pool of integrin receptors including *α*_6_ *β*_1_, *α*_2_ *β*_1_, *α*_D_ *β*_2_, *α*_M_ *β*_2_, *α*_v_ *β*_3_, *α*_v_ *β*_5_, and *α*_IIb_ *β*_3_ [8–14]. For instance, CCN1 promotes cell adhesion through *α*_V_ *β*_3_ in endothelial cells [12], through *α*_IIb_ *β*_3_ in platelets [14], through *α*_6_ *β*_1_ in fibroblasts [13], and through *α*_M_ *β*_2_ in monocytes [11]. On the other hand, while CCN1 is known to adopt different receptors to diversify its signaling in the same tissue, as best exemplified in fibroblasts, where it induces cell adhesion through *α*_6_ *β*_1_ and heparan sulfate proteoglycans, cell migration through *α*_v_ *β*_5_, and cell proliferation through *α*_v_ *β*_3_ [15], it was also shown to activate both NF-*κ*B [16] and MAPK signaling [17] in breast cancer cells through the same receptor, namely *α*_v_ *β*_3_.

Overexpression of CCN1 has been found to activate *β*-catenin translocation in several cell systems including glioma [18], non-small-cell lung cancer cells [19], and gastric epithelial cells [20], whereas *β*-catenin translocation can in turn promote CCN1 expression through Wnt signaling [21]. Over the last few years, a growing amount of data have been generated about CCN1 signaling in mesenchymal cells [8, 13, 15, 22, 23], while its activities in epithelial cells remain largely unknown. In particular, CCN1 is one of the most highly expressed proteins in the esophageal epithelium and has been associated with various pathological conditions, including ESCC [24], Barrett's esophagus, and esophageal adenocarcinoma [25]. Moreover, we recently showed that CCN1, which is upregulated under acidic conditions [26], mediates acid-induced esophageal epithelial-mesenchymal cellular transformation [27], suggesting a critical role in esophageal malignancy.

## 2. Materials and Methods

### 2.1. Cell Culture, Transfection, and Treatment

Human esophageal squamous epithelial Het-1A cells (American Type Culture Collection, Manassas, VA) and esophageal squamous carcinoma OE21 cells (Sigma-Aldrich, St. Louis, MO) were maintained in BEGM plus supplements and RPMI plus 10% serum (Lonza, Walkersville, MD), respectively. To knock down CCN1, cells were transfected with shRNA against CCN1 in pRFP-C-RS vector (Origene, Rockville, MD), which constitutively expresses red fluorescent protein (RFP) in the transfected cells. Prior to each experiment, cells were serum-starved overnight and then treated with either vehicle (control) or recombinant human CCN1 protein (PeproTech, Rocky Hill, NJ) at 100 ng/mL for an indicated period of time.

### 2.2. Gene Expression Analysis

Total RNA was extracted from Het-1A cells using Trizol reagent (Invitrogen, Carlsbad, CA) and further purified using RNeasy kit (QIAGEN, Valencia, CA). Probe was synthesized through reverse transcription using a GEArray kit (SABiosciences, Frederick, MD) and labeled with Biotin-16-dUTP (Roche, Mannheim, Germany) following the manufacturer protocol. We used GEArray Q series microarray membranes (SABiosciences) that contain 96 cDNA sequences for extracellular matrix and adhesion molecules to screen integrins. Briefly, the microarray membrane was prehybridized with the denatured salmon sperm DNA solution (Sigma-Aldrich) in a hybridization oven (Fisher Scientific, Pittsburgh, PA) at 60°C for one hour and then hybridized with the labeled probe at the same temperature overnight. The membrane was washed in 2X SSC plus 1% SDS for 2 × 15 min at 60°C and then in 0.1X SSC plus 0.5% SDS for additional 2 × 15 min. The signal was developed using a chemiluminescent kit (SABiosciences), captured on X-ray film, and quantified using ImageQuant 3.3 software (GE Life Sciences, Piscataway, NJ).

Expression of selected genes was also confirmed by real-time PCR analysis. Briefly, 0.8 *μ*g RNA was used as templates to generate cDNA through reverse transcription reaction in MyCycler (Bio-Rad, Hercules, CA) following standard protocol (25°C/10 min-55°C/30 min-85°C/5 min-4°C/5 min). *β*-actin was included as internal control. Real-time PCR was performed in iCylcer (Bio-Rad) following two-step manufacturer protocol. Data were analyzed using the ΔΔC_t_ method based on at least 3 independent experiments. Briefly, ΔC_t_ was calculated by subtracting the C_t_ value of *β*-actin from the C_t_ value for each gene of interest; then ΔΔC_t_ was calculated by subtracting the ΔC_t_ of the control from the ΔC_t_ of the treatment for each gene of interest; finally the fold change was calculated using the formula: Fold Change =2^(−ΔΔC_t_)^.

### 2.3. Protein Expression Analysis

Protein isolation and Western blotting were done as described in our previous studies [20, 28]. Briefly, equal amounts of protein were separated by SDS-PAGE and transferred to nitrocellulose membrane. Membranes were blocked in 5% milk in phosphate-buffered saline with 1% Tween-20 and incubated in primary antibody for 2 hrs and in HRP-conjugated secondary antibody for 45 minutes. The signal was developed using a chemiluminescent kit, captured on X-ray film, and quantified using ImageQuant 3.3 software. The following primary antibodies were used: rabbit anti-CCN1 and goat anti-integrin *α*_11_ (Santa Cruz Biotech, Santa Cruz, CA); mouse anti-phosphoserine/threonine (BD Biosciences, San Diego, CA); mouse anti-active-*β*-catenin (Millipore, Billerica, MA); rabbit anti-*β*-catenin (Abcam); mouse anti-*β*-actin (Sigma-Aldrich).

For immunoprecipitation, as described in our previous studies [20, 28], 100 *μ*g total protein was incubated with 1 *μ*g rabbit anti-*β*-catenin antibody for 2 hours at 4°C on rocker prior to addition of agarose A beads (Millipore), and then the total mixture was incubated again overnight on rocker at 4°C. Beads were precipitated and washed three times with lysis buffer prior to final resuspension in 2X sample buffer. Samples were boiled for 5 minutes and then separated on 7% SDS-PAGE gels, transferred to nitrocellulose membrane, and analyzed by Western blotting as described.

### 2.4. Immunofluorescence Microscopic Analysis

Cells were grown in Lab-Tek chamber slides (Nalge, Naperville, IL). After an appropriate treatment as indicated, cells were fixed for 10 min in cold acetone and incubated in serum-free Protein Block (Dako, Carpentaria, CA) for 30 min to eliminate possible nonspecific antibody binding. Then, some slides were probed with mouse anti-*β*-catenin antibody (1 : 500; Abcam) for 2 hours to detect *β*-catenin expression and localization, and control slides were incubated with a preimmune serum to monitor the specificity of the antibody. FITC-conjugated secondary antibody (1 : 1000; Abcam) was used to detect the signal.

### 2.5. Immunohistochemistry

Human esophageal tissue slides (Capital Biosciences, Rockville, MD) were deparaffinized in Histoclear solution (National Diagnostics, Atlanta, GA) and rehydrated in a series of ethanol. Antigens were retrieved by repeatedly microwave boiling in 0.01 M sodium citrate buffer (pH 6.0). Endogenous peroxidase was blocked in Dako reagent for 10 minutes. Slides were then incubated with the following primary antibodies at 4°C overnight: rabbit anti-CCN1 (1 : 200), rabbit anti-*β*-catenin (1 : 500), and goat anti-integrin *α*_11_ (1 : 50). The LSAB + kit (Dako) was used to develop a signal.

### 2.6. Detection of Protein-Protein Interactions by Proximity Ligation

Protein-protein interactions were analyzed *in situ* using the duolink PLA-probe detection system (Olink Bioscience/Axxora LLC, San Diego, CA). Briefly, cells were cultured on cover slips, fixed in cold acetone, blocked and incubated in a primary antibody as described above for cell staining, followed by 2-hour incubation in rabbit and goat PLA PLUS and PLA MINUS probes, 15-minute hybridization, 15-minute ligation, 90-minute amplification, and 60-minute detection according to the manufacturer protocol. Goat anti-integrin *α*_11_ was coupled with rabbit anti-CCN1. To maximize signal strength, samples were incubated in primary antibody overnight (4°C) and PLA probes were used at a 1/2.5 dilution. All steps after primary antibody incubation were carried out at 37°C. Nuclei were counterstained with Hoechst 33342 (blue). This system uses special secondary antibodies containing a short DNA strand (PLA probes), which can hybridize and form a circularized oligo that can be amplified and detected as a discrete fluorescent dot (individual interaction between two molecules) when two opposite probes come in close proximity (less than 40 nm) [29].

### 2.7. Integrin Functional Blockage

Cells were serum starved overnight in basal medium. Where indicated, cells were then preincubated with either diluted preimmune serum (control) or the antibody against the extracellular domain of integrin *α*_11_ at 50 *μ*g/mL in fresh basal medium for 3 hours. Recombinant CCN1 protein (100 ng/mL) was then added to the medium, and cells were incubated again overnight. Nontreated cells and CCN1-treated cells cultured in the absence of antibody pretreatment were included as negative and positive control respectively. The effect of integrin blockage on CCN1 signaling was assessed by *β*-catenin nuclear translocation through immunofluorescence microscopic analysis as well as by *β*-catenin activation through immunoprecipitation and Western blot analysis as described above.

### 2.8. Statistical Analysis

All experiments were performed at least in triplicates. All numerical data were analyzed by single-classification one-way ANOVA, and *P* < 0.05 was considered significant.

## 3. Results

### 3.1. Knockdown of CCN1 in ESCC Curtails *β*-Catenin Expression and Translocation

**Figure 1:**
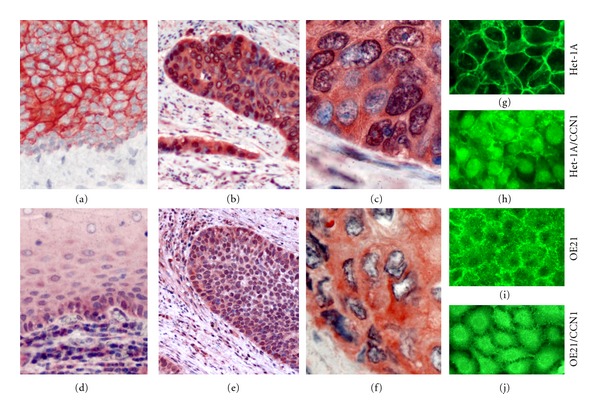
CCN1 induces *β*-catenin translocation in esophageal epithelial cells. (a) In normal esophageal mucosa, *β*-catenin is localized to the cell membrane (250x). (b) In ESCC tumor tissue, *β*-catenin expression is highly elevated (250x). (c) *β*-catenin in ESCC tissue is mainly localized in the cytoplasm and some appears in the nucleus as well (600x). (d) In normal esophageal mucosa, CCN1 is primarily expressed in the basal cells (250x). (e) CCN1 expression is elevated in ESCC tumor tissue (250x). (f) CCN1 is localized to both cellular and extracellular matrix in ESCC tumor tissue (600x). (g) In Het-1A cells, *β*-catenin is neatly confined to intercellular connections. (h) Under CCN1 treatment, *β*-catenin in Het-1A cells accumulates in the cytoplasm and the nucleus. (i) In OE21 cells, *β*-catenin appears in both the cytoplasm and the cell membrane. (j) Under CCN1 treatment, *β*-catenin in OE21 cells becomes more concentrated in the nuclear area.

Comparing to normal esophageal mucosa in which *β*-catenin was neatly localized to the cell membrane (Figures 1(a) and 1(g)), *β*-catenin in ESCC was not only elevated but also mostly dissociated from the membrane and accumulated in the cytoplasm, and some even appeared in the nucleus (Figures 1(b), 1(c) and 1(i)). CCN1, correspondingly, was heavily elevated in ESCC (Figures 1(e) and 1(f)), compared to normal esophageal mucosa in which CCN1 was mainly confined to the basal cells and gradually faded away towards the mucosal surface (Figure 1(d)), indicating an involvement in cell proliferation. After an incubation with recombinant human CCN1 protein at 100 ng/mL for 6 hours, normal esophageal Het-1A cells displayed a heavy accumulation of *β*-catenin in both the cytoplasm and the nucleus (Figure 1(h)), similar to what was observed in ESCC tumor tissue, whereas in ESCC OE21 cells in which *β*-catenin was originally expressed in the cytoplasm, adding exogenous CCN1 made it more concentrated in the nuclear areas (Figure 1(j)).

**Figure 2:**
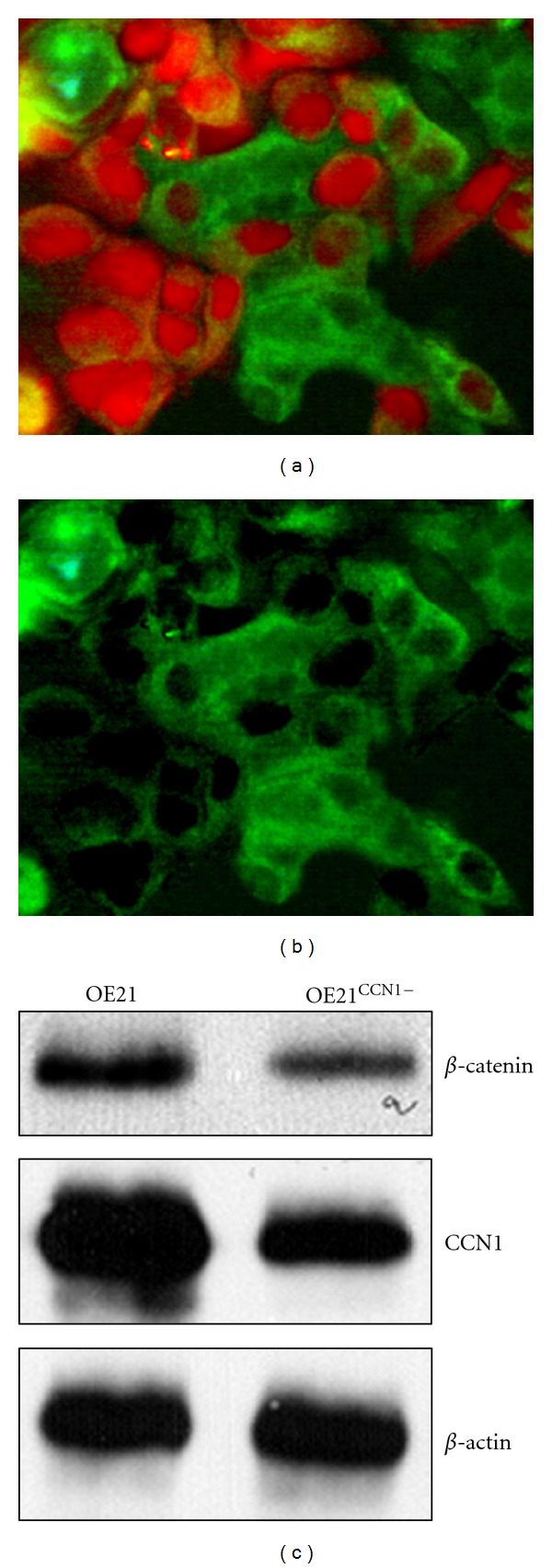
Knockdown of CCN1 in OE21 ESCC cells (OE2^CCN1−^) reduces the level of *β*-catenin. (a) Under a dual filter, the shRNA transfected cells display bright red fluorescence due to expression of RFP, while *β*-catenin is identified in green by FITC-conjugated antibody. (b) In the same view under FITC filter, the shRNA transfected cells display low level of *β*-catenin compared to nontransfected cells. (c) Western blot analysis shows reduced expression of *β*-catenin in OE21^CCN1−^ cells.

To know whether *β*-catenin elevation and translocation in ESCC are associated with CCN1 upregulation, we transfected OE21 cells with RFP-conjugated shRNA against CCN1, and then we stained the cells for *β*-catenin and detected it with a FITC-conjugated secondary antibody. In those transfected cells (easily identified by the bright red fluorescence), *β*-catenin level was decreased and its distribution was more confined to the cell membrane (Figures 2(a) and 2(b)), indicating that CCN1 is responsible (at least partially if not all) for *β*-catenin elevation and translocation in ESCC. Western blot analysis also confirmed the downregulation of *β*-catenin in OE21 cells by CCN1 knockdown (Figure 2(c)).

### 3.2. CCN1 Upregulates Expression of ITGA11 in Esophageal Epithelial Cells

**Figure 3:**
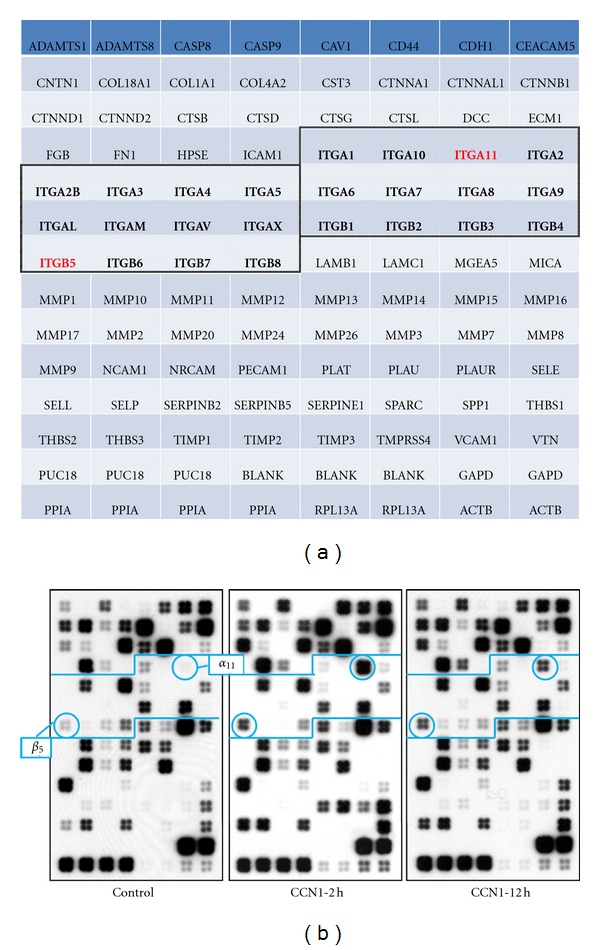
CCN1 upregulates gene expression of ITGA11 and ITGB5 in esophageal epithelial cells. Cells were cultured in the absence (control) or presence of CCN1 for the times indicated. Total RNA was isolated and reverse-transcribed with Biotin-16-dUTP to generate cDNA probes, which were then hybridized to the cDNA microarrays. (a) Array layout with integrins is marked in bold. Integrins further analyzed in the study are in red. (b) Gene expression profile with marked (circled) integrins of interest. ITGA11 and ITGB5 are upregulated by CCN1.

Based on what we discussed previously, that is, CCN1 signals through different integrin receptors in a cell-dependent manner, we were interested to know what integrin(s) mediates CCN1-activated *β*-catenin translocation in esophageal epithelial cells. Since CCN1 signaling in esophageal epithelial cells has never been studied, we decided to screen the integrin library to look for candidate(s). Out of 16 *α*- and 8 *β*-integrins examined (Figure 3(a)), only two responded to CCN1 treatment significantly: ITGA11 was increased by 143.98-fold and 61.78-fold at 2 and 12 hours respectively, and ITGB5 was increased by 5.68-fold and 4.71-fold correspondingly (all *P* < 0.01). Since integrin *β*_5_ is a known receptor of CCN1 [13, 15], integrin *α*_11_ was chosen for further investigation.

**Figure 4:**
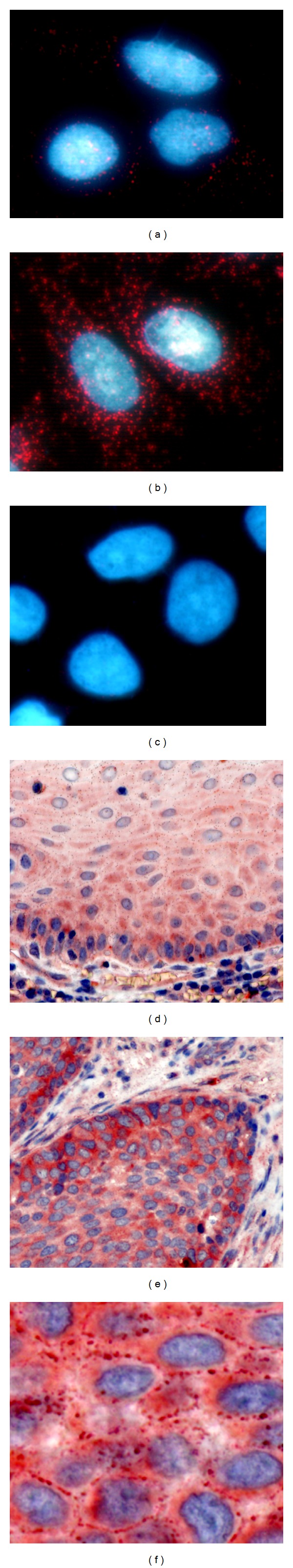
Integrin *α*_11_ is elevated in ESCC tumor tissue, and CCN1 has direct interaction with integrin *α*_11_ esophageal epithelial cells. (a) Endogenous CCN1 interacts with integrin *α*_11_ in Het-1A cells although signals are weak (control). (b) After Het-1A cells were treated with CCN1 for 6 hours, the signals reflecting CCN1 and integrin *α*_11_ interaction were greatly increased. (c) Adding CCN1 antibody to the cell culture eliminated the signal of CCN1-integrin *α*_11_ interaction. (d) Integrin *α*_11_ expression in normal esophageal mucosa (250x). (e) Integrin *α*_11_ is elevated in ESCC tumor tissue (250x). (f) Integrin *α*_11_ is mainly localized to the cell membrane in ESCC tumor (600x).

To determine whether there is a direct interaction between CCN1 and integrin *α*_11_, we used a novel protein-protein interaction technique (DUOLINK system). Nowadays, more and more studies use this technology to visualize *in situ* ligand-receptor binding [30, 31]. In the CCN1-treated Het-1A cells, massive signals (red dots) were detected, reflecting active interactions between these two molecules (Figure 4(b)). However, some signals were also detected in the nontreated cells (Figure 4(a)), indicating the existence of natural interactions between endogenous CCN1 and integrin *α*_11_. On the other hand, blocking CCN1 with excessive antibody diminished the signal, confirming the specificity of the interaction (Figure 4(c)).

### 3.3. Integrin *α*_11_ Is Overexpressed in ESCC

Based on our results shown above, that is, CCN1 is elevated in ESCC and it can increase integrin *α*_11_ expression, we predicted that integrin *α*_11_ expression would likely be elevated in ESCC. To test our hypothesis, we compared integrin *α*_11_ expression in ESCC tumor tissue with normal esophageal mucosa (Figure 4(d)) by immunohistochemistry. Integrin *α*_11_ was found highly elevated in ESCC (Figure 4(e)), and some was neatly localized to the cell membrane (Figure 4(f)).

### 3.4. Integrin *α*_11_ Mediates CCN1-Induced *β*-Catenin Translocation

**Figure 5:**
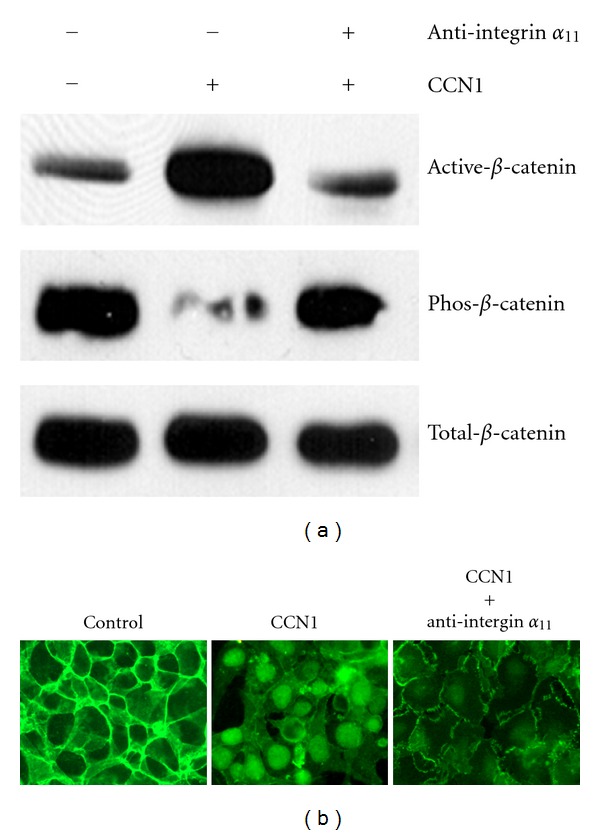
Integrin *α*_11_ mediates CCN1-induced *β*-catenin activation and translocation. (a) Het-1A cells were pretreated with either preimmune serum (control) or anti-integrin *α*_11_ antibody cultured in the absence (−) or presence (+) of CCN1 overnight. *β*-catenin was pulled down from the cells lysates through immunoprecipitation, subsequently analyzed by Western blot for expression of total *β*-catenin, active *β*-catenin (dephosphorylated at Ser37 or Thr41), and Ser/Thr-phosphorylated *β*-catenin. (b) Immunofluorescence microscopic analysis shows that blocking integrin *α*_11_ with a specific antibody prevents CCN1 to induce *β*-catenin translocation.

Naturally, next we wanted to know whether integrin *α*_11_ was involved in CCN1-induced *β*-catenin translocation in esophageal cells. We treated Het-1A cells with recombinant CCN1 for 12 hours, the level of the active form of *β*-catenin (dephosphorylated on serine-37 or threonine-41) was increased by 109.1% (*P* < 0.01), which was confirmed by reprobing the blots for phosphorylated serine/threonine (Figure 5(a)). However, when excessive anti-integrin *α*_11_ antibody was added to the cell culture, CCN1 failed to induce activation of *β*-catenin (Figure 5(a)). These results were also confirmed by immunofluorescence microscopic analysis, where CCN1 failed to induce *β*-catenin translocation in the presence of integrin *α*_11_ antibody and *β*-catenin remained predominantlyinthe membrane instead (Figure 5(b)), suggesting that integrin *α*_11_ is required to transduce CCN1-mediated *β*-catenin translocation in esophageal epithelial cells.

## 4. Discussion

While CCN1 is best known for its angiogenic activity [32, 33], its role in epithelial cells is largely unknown. Our recent study demonstrates that CCN1 can induce a transient epithelial-mesenchymal transition during gastric ulcer healing to facilitate the process of re-epithelialization [20]. A similar effect was also noted in esophageal epithelial cells [26], suggesting a protective part for CCN1 in normal epithelial cells. In cancers, on the other hand, CCN1 is like a double-edged sword: in gastric cancer it was found to enhance tumorigenicity [34], while in squamous lung carcinoma it served the opposite role [35]. Furthermore, the expression level of CCN1 is highly dependent on the type and the stage of the cancer. Elevation of CCN1 has been found in breast cancer [16], pancreatic cancer [36], and gliomas [18], while in endometrial cancer [37] and lung carcinoma [35], CCN1 level is decreased. More complex or even conflicting results have been reported in some other cancers. For instance, in 1998, CCN1 downregulation was reported in prostate cancer [38], while more recent studies showed the opposite results [36, 37]. In colorectal cancer, CCN1 mRNA was elevated compared with normal colon, but it was reduced in more advanced stages [38]. A similar trend was also noted in our study on esophageal adenocarcinoma development from gastroesophageal reflux disease, in which CCN1 was highly elevated in acid reflux condition and continued its overexpression at the lower level during metaplastic transformation, but it dropped in the advanced adenocarcinoma [39]. Recently, CCN1 upregulation has been reported in ESCC [24, 40, 41], in which high expression of CCN1 was noted in association with poor survival of ESCC patients [24].

In our previous study, we found that CCN1 can induce a transient *β*-catenin translocation in normal gastric epithelial cells [20] and thereby promotes ulcer healing process by accelerating epithelialization. Once the wound is healed, *β*-catenin can return to its usual level and function. In this current study, we showed that *β*-catenin elevation and translocation in ESCC tumor cells are dependent on high level of CCN1. Knockdown of CCN1 can reduce *β*-catenin. *β*-catenin is a key player in Wnt signaling, and its activation/translocation can lead to expression of a number of growth-related genes. Some of these genes are oncogenic (e.g., c-myc, c-jun), which can contribute to cancer development. In addition, *β*-catenin translocation can also destabilize intercellular connection, facilitating cancer metastasis. CCN1 just happens to be one of Wnt signaling targets. Therefore, these two molecules can form a CCN1-*β*-catenin-CCN1 loop to amplify the signal, thereby to accelerate wound healing or to promote oncogenesis, depending on the context. Further studies on the relationship of these two proteins would be very rewarding.

CCN1 functions through different integrin receptors in a tissue-dependent manner. Out of 26 known integrins, at least 8 have been found to mediate CCN1 actions in various cells. The context dependency of CCN1 is reflected in its receptor selection. Here we showed that integrin *α*_11_ is required for CCN1 to induce *β*-catenin translocation in esophageal epithelial cells. ITGA11, the gene encoding integrin *α*_11_ protein, was cloned and sequenced a decade ago by two independent groups [42, 43]. Both groups examined the expression pattern of integrin *α*_11_ in adult tissue and found that it was mainly expressed in muscular-rich organs such as uterus, heart, and skeletal muscle. They also identified moderate expressions of integrin *α*_11_ in the gastrointestinal tract, including the stomach, the intestine, and the colon, although the esophagus was not examined. Up to date, there are less than 20 studies on ITGA11 published, most of which focus on mesenchymal tissues, where integrin *α*_11_ mediates such processes as migration of embryonic fibroblasts [44], tooth eruption [45], and corneal development and disease [46]. On the other hand, the expression and function of integrin *α*_11_ in epithelial cells have never been fully investigated. In this study, we showed that ITGA11 is normally expressed at minimal levels in esophageal epithelial cells, but it can be dramatically upregulated by CCN1, a feedback mechanism that CCN1 is known to employ for some of its receptors in other tissues. Consistent with the possible role of integrin *α*_11_ as a CCN1 receptor, a direct interaction between these two proteins was demonstrated, and blocking integrin *α*_11_ function prevented CCN1 from eliciting its downstream signals in these cells. 

In conclusion, our study has demonstrated that the elevation and translocation of *β*-catenin in ESCC require high level of CCN1, and integrin *α*_11_, which has so far been studied mostly in mesenchymal tissues, mediates this process. Given the importance of nuclear *β*-catenin in cancer progression [47, 48], particularly in ESCC [49, 50] and esophageal adenocarcinoma [51, 52], our findings could be one step closer to better understanding of the molecular progression of esophageal malignancy. 

## List of Abbreviations

ESCC: esophageal squamous cell carcinoma.
